# PAMPs and DAMPs as triggers for DIC

**DOI:** 10.1186/s40560-014-0065-0

**Published:** 2014-12-31

**Authors:** Takashi Ito

**Affiliations:** Department of Emergency and Critical Care Medicine, Kagoshima University Graduate School of Medical and Dental Sciences, Kagoshima, Japan; Department of Systems Biology in Thromboregulation, Kagoshima University Graduate School of Medical and Dental Sciences, Kagoshima, Japan

**Keywords:** Immunothrombosis, Pathogen-associated molecular patterns (PAMPs), Damage-associated molecular patterns (DAMPs), Tissue factor, Neutrophil extracellular traps (NETs), Disseminated intravascular coagulation (DIC)

## Abstract

Thrombosis is generally considered harmful because it compromises the blood supply to organs. However, recent studies have suggested that thrombosis under certain circumstances plays a major physiological role in early immune defense against invading pathogens. This defensive role of thrombosis is now referred to as immunothrombosis. Activated monocytes and neutrophils are two major inducers of immunothrombosis. Monocytes and neutrophils are activated when they detect pathogen-associated molecular patterns (PAMPs) and damage-associated molecular patterns (DAMPs). Detection of PAMPs and DAMPs triggers tissue factor expression on monocytes and neutrophil extracellular trap (NET) release by neutrophils, promoting immunothrombosis. Although tissue factor-mediated and NET-mediated immunothrombosis plays a role in early host defense against bacterial dissemination, uncontrolled immunothrombosis may lead to disseminated intravascular coagulation.

## Introduction

Blood must be maintained in a fluid state under physiologic conditions, but then change to a solid state after vascular injury. This balancing act is accomplished by platelets, coagulation factors, anticoagulant factors, fibrinolytic factors, endothelial cells, and possibly leukocytes, which all support the dynamic equilibrium that provides proper blood flow [1]. Disruption of this well-regulated balance leads to pathologic conditions, such as thrombosis and bleeding.

## Review

### Basic mechanisms of hemostasis

**Figure 1:**
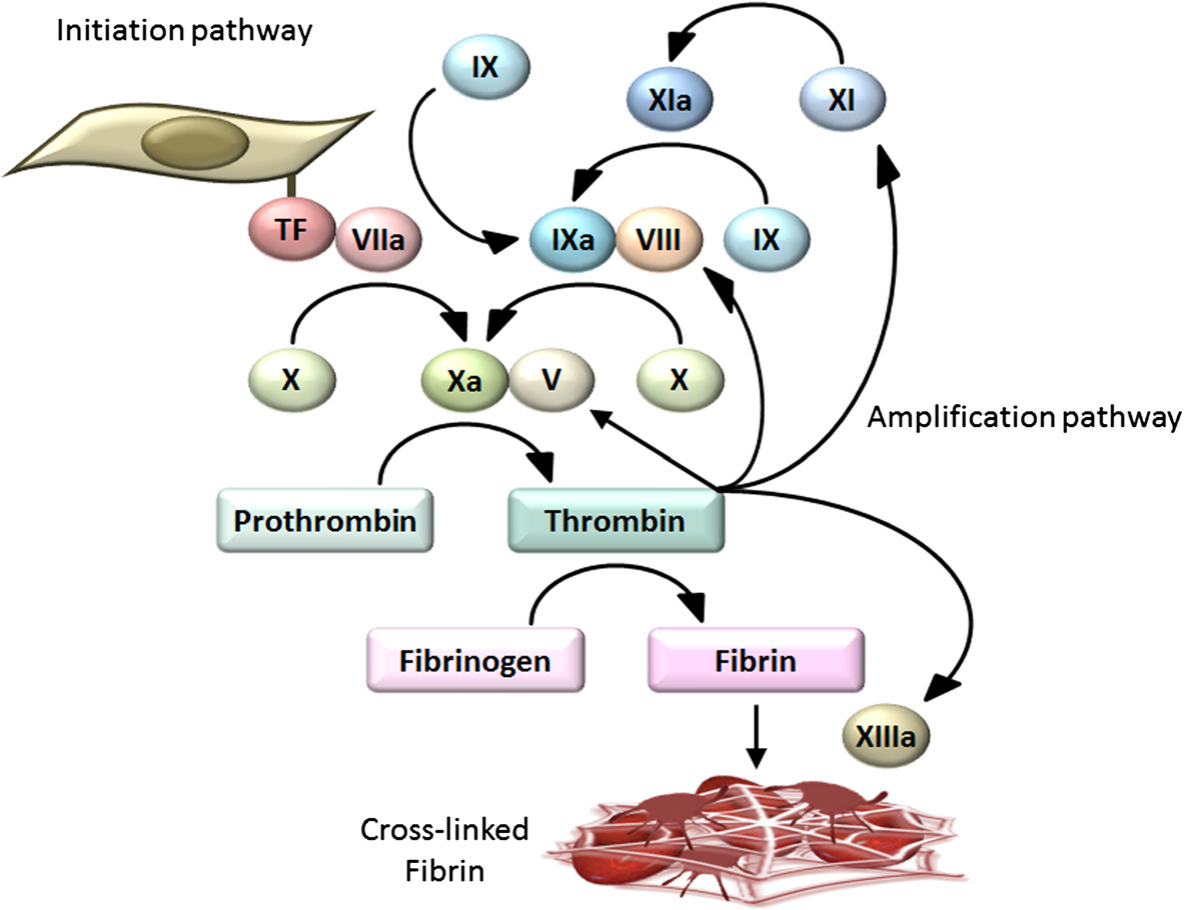
**Basic mechanisms of coagulation.** Coagulation factor VII searches for sites of vascular damage where subendothelial tissue factor is exposed. Tissue factor is expressed on the surface of fibroblasts and pericytes in the subendothelial space. Binding of coagulation factor VIIa to tissue factor results in a cascade of blood-clotting reactions, leading to thrombin generation (the initiation pathway). Once small amounts of thrombin are generated in this pathway, thrombin plays a crucial role in the amplification and propagation phases of coagulation by activating coagulation factors V, VIII, and XI (the amplification pathway). This leads to a burst of additional thrombin generation, which is essential for forming sufficient fibrin and sealing the sites of vascular damage. Coagulation factor XIII then crosslinks fibrin fibers, a fundamental process for stabilizing fibrin clots. Contact activation of coagulation factor XII, another important trigger of coagulation in laboratory tests, is not considered essential for hemostasis.

Platelets and coagulation factors are two major players in hemostasis. Platelets and coagulation factors circulate in the blood and become activated at sites of vascular damage. Platelets monitor vascular damage using cell-surface sensors for subendothelial collagen and von Willebrand factor bound to collagen. Engagement of the subendothelial matrix by platelets results in a sequence of reactions comprising platelet adhesion, activation, and aggregation, leading to platelet thrombus formation [2]. Coagulation factors, more specifically coagulation factor VII, search for sites of vascular damage where subendothelial tissue factor is exposed. Binding of coagulation factor VIIa to tissue factor results in a cascade of blood-clotting reactions, leading to thrombin generation and subsequent fibrin deposition at sites of vascular damage (Figure 1). Platelet thrombus formation and fibrin deposition occur concomitantly as thrombin activates platelets, and activated platelets expose phosphatidylserine on their membrane surface to provide a scaffold for blood-clotting enzyme complexes [3].

In the hemostatic system, thrombin generation is triggered by the factor VIIa-tissue factor complex, an inducer of the so-called extrinsic pathway. Once small amounts of thrombin are generated in this pathway, thrombin plays a crucial role in the amplification and propagation phases of coagulation, the so-called intrinsic pathway, by activating coagulation factors V, VIII, and XI (Figure 1) [1]. This leads to a burst of additional thrombin generation, which is essential for forming sufficient fibrin and sealing the sites of vascular damage. Coagulation factor XIII then crosslinks fibrin fibers, a fundamental process for stabilizing fibrin clots. Contact activation of coagulation factor XII, another important trigger of coagulation in laboratory tests, is not considered essential for hemostasis because hereditary deficiencies in factor XII are not associated with abnormal bleeding [4,5]. However, factor XII might be involved in pathological thrombosis [6-8] and could be a unique drug target suitable for preventing thrombosis without affecting normal hemostasis [5].

**Figure 2:**
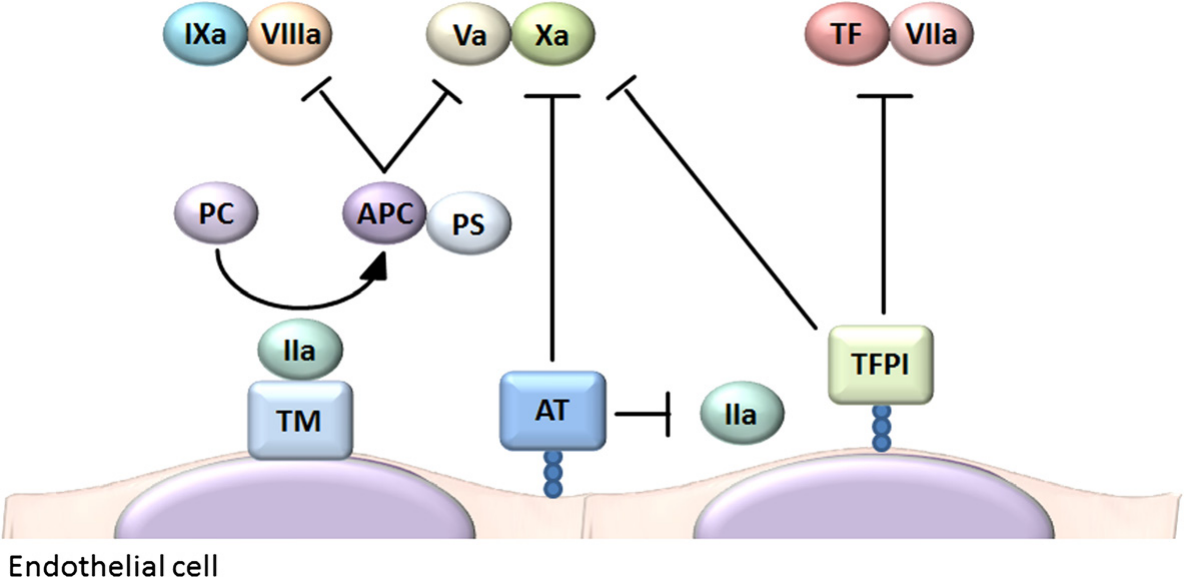
**Anticoagulant properties of endothelial cells.** Endothelial cells express several anticoagulants, including thrombomodulin (TM), tissue factor pathway inhibitor (TFPI), and heparan sulfate. Upon binding to TM, thrombin loses its ability to activate platelets, fibrinogen, and coagulation factors V, VIII, XI, and XIII. Furthermore, the thrombin-TM complex activates protein C, which in turn stops thrombin generation by inactivating coagulation factors Va and VIIIa. Endothelial cells also synthesize and display heparan sulfate proteoglycans on their surface, which bind to TFPI and antithrombin (AT), inhibiting the factor VIIa-tissue factor complex, factor Xa, and thrombin activity. *IIa* thrombin, *PS* protein S.

The propagation of a hemostatic plug can be terminated when it reaches intact endothelium. Endothelial cells express several anticoagulants, including thrombomodulin (TM), tissue factor pathway inhibitor (TFPI), and heparan sulfate (Figure 2) [9,10]. Upon binding to TM, thrombin loses its ability to activate platelets, fibrinogen, and coagulation factors V, VIII, XI, and XIII [11]. Furthermore, the thrombin-TM complex activates protein C, which in turn stops thrombin generation by inactivating coagulation factors Va and VIIIa. Endothelial cells also synthesize and display heparan sulfate proteoglycans on their surface, which bind to TFPI and antithrombin (AT), inhibiting the factor VIIa-tissue factor complex, factor Xa, and thrombin activity [10]. Thus, endothelial cells play a role in regulating the spatial localization of hemostatic plugs. Disruption of this well-regulated balance leads to thrombus formation inside blood vessels (i.e., thrombosis) [1].

### Immunothrombosis

Microvascular thrombosis is a frequent complication of critical illness conditions, such as sepsis, trauma, and malignancy [12]. Thrombosis is generally considered harmful because it compromises the blood supply to organs. However, recent studies have suggested that thrombosis under certain circumstances plays a major physiological role in immune defense [13,14]. This defensive role of thrombosis is now referred to as immunothrombosis [13].

Thrombus formation and innate immunity are closely linked [15]. Upon injury, multicellular organisms face two major crises: bleeding and infection. To overcome these crises, multicellular organisms have developed hemostatic systems and immune systems. In horseshoe crabs, a single hemocyte type circulates in their open circulatory system and plays significant roles in both hemostasis and innate immunity [16]. The hemocytes release procoagulant serine protease zymogens when they detect lipopolysaccharide (LPS) on their surface. The activation of these zymogens triggers the coagulation cascade, which ultimately converts coagulogen into insoluble coagulin gels [17]. Coagulin clots are important not only for sealing injured sites but also for trapping invading pathogens and supporting antimicrobial defense. Thus, the hemocytes of horseshoe crabs can detect and respond sensitively to LPS, maintaining hemostasis and host defense against invading pathogens. Horseshoe crab hemocytes are now used for laboratory measurements of endotoxins.

Coagulation systems in mammals also play important roles in immune defense. Fibrinogen-deficient mice display impaired cytokine production, suppressed neutrophil recruitment, increased bacterial burden, and increased mortality after bacterial inoculation [18,19]. Furthermore, mice pretreated with anticoagulants, such as coumadin or hirudin, also display increased bacterial burden and mortality following bacterial inoculation [18,20]. These phenotypes indicate protective roles of coagulation systems during early host defense against bacterial dissemination. In humans, coagulation systems are also activated during infection [21]. However, it remains to be determined whether anticoagulant therapy improves or worsens the clinical outcomes of patients with infectious diseases.

Platelets have important roles in fighting infections. Upon bacterial infection, platelets rapidly accumulate on the surface of bacteria caught by Kupffer cells [22]. The platelet-mediated encasement of bacteria restricts their escape from Kupffer cells. This event precedes leukocyte recruitment and contributes to early host defense against infection in mice. Platelets are able to release antimicrobial molecules and proinflammatory mediators, which may further support host defense against infection [23]. In humans, thrombocytopenia is increasingly recognized as an independent risk factor for serious infections [23]. Furthermore, antiplatelet therapy may be associated with increased incidence of community-acquired pneumonia [24], although it may also be associated with better outcomes in patients with severe infections [25,26]. These observations suggest that platelets may be important in early host defense against invading pathogens before infectious diseases develop but may be deleterious if infections progress to severe forms with organ failure.

**Figure 3:**
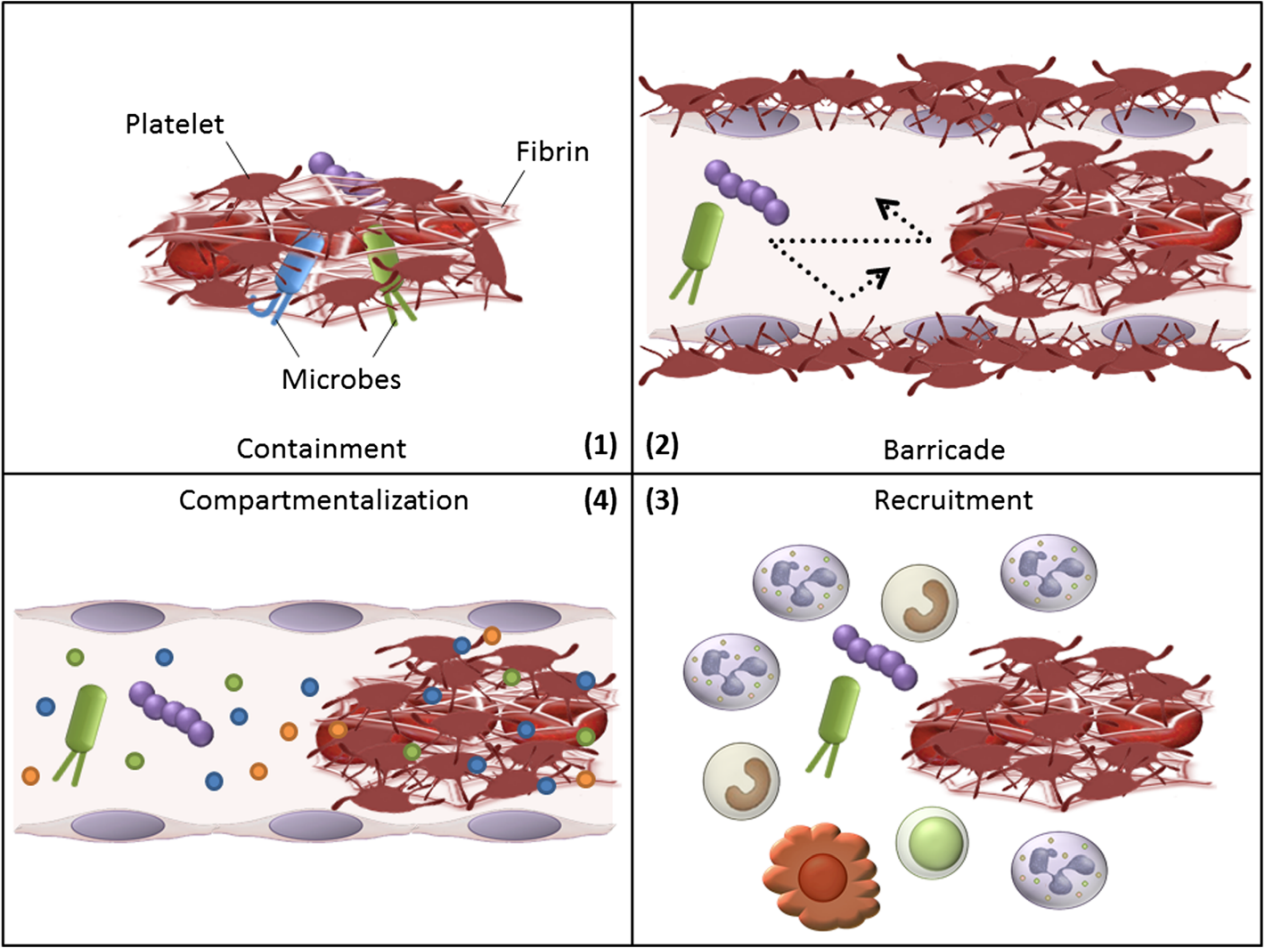
**Four mechanistic models explaining how immunothrombosis provides protection against invading pathogens.** (1) Immunothrombosis limits microbial dissemination by containing microbes within thrombi. (2) Thrombi form protective barricades inside and/or around blood vessels that limit microbial movement in and out of the vessels. (3) Fibrin, fibrinogen, and fibrin/fibrinogen degradation products promote recruitment and activation of leukocytes, such as neutrophils and macrophages, coordinating cellular immune responses to pathogens at sites of infection. (4) Intravascular thrombi yield a distinct compartment where antimicrobial peptides are concentrated and have increased opportunities to come into contact with pathogens.

To date, four mechanistic models have been proposed for how immunothrombosis provides protection against invading pathogens (Figure 3) [13]. First, immunothrombosis limits microbial dissemination by retaining microbes within thrombi. In this regard, coagulation factor XIII crosslinks bacteria to fibrin fibers, leading to immobilization and killing of bacteria inside the clot [27]. Second, thrombi form protective barricades inside and/or around blood vessels that limit microbial movement in and out of the vessels [20]. Third, fibrin, fibrinogen, and fibrin/fibrinogen degradation products promote recruitment and activation of leukocytes, such as neutrophils and macrophages, coordinating cellular immune responses to pathogens at sites of infection [28]. Fourth, intravascular thrombi yield a distinct compartment where antimicrobial peptides are concentrated and have increased opportunities to come into contact with pathogens. Antimicrobial peptides can be released not only by leukocytes but also by platelets and coagulation systems during the process of immunothrombosis [23,29].

### Triggers for immunothrombosis

**Figure 4:**
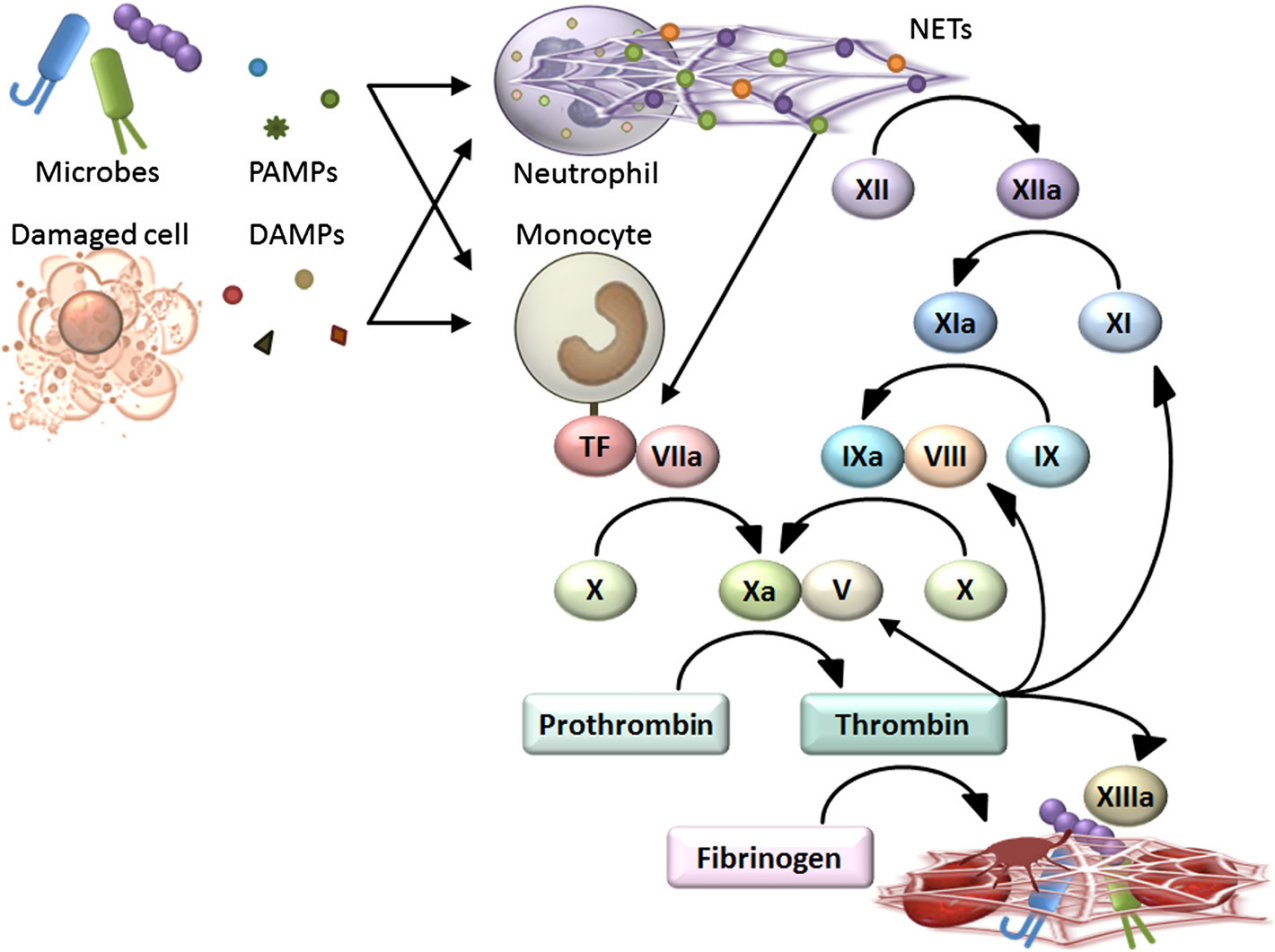
**Triggers for immunothrombosis.** Detection of PAMPs and DAMPs triggers NET release by neutrophils and tissue factor expression on monocytes, promoting immunothrombosis. NETs are able to activate coagulation factor XII, inactivate anticoagulant TFPI, and provide a scaffold for platelet binding and aggregation, all of which promote thrombus formation. A part of monocyte-associated tissue factor is released in the form of microparticles and delivered into developing thrombi.

What are the triggers for immunothrombosis? During the course of infections, platelets and coagulation factors can become activated even in the absence of contact with subendothelial collagen and tissue factor. It is now widely believed that instead of subendothelial collagen and tissue factor, neutrophils and monocytes could serve as the triggers for immunothrombosis (Figure 4) [13].

Monocytes are a potential source of tissue factor in circulating blood [30]. In contrast to subendothelial tissue factor constitutively expressed on fibroblasts and pericytes, monocyte-associated tissue factor expression is normally very low and increases in response to pathogen stimuli [31]. Genetic reduction of tissue factor expression on leukocytes reduces LPS-induced thrombin generation [32,33], suggesting that tissue factor on leukocytes activates coagulation in response to pathogen stimuli. Furthermore, low tissue factor expression results in bacterial dissemination and poor outcomes following bacterial inoculation [18]. These findings indicate that monocyte-associated tissue factor is an important inducer of immunothrombosis.

Neutrophils and neutrophil extracellular traps (NETs) are other inducers of immunothrombosis [13]. In response to pathogen stimuli, neutrophils initiate a program involving rearrangement of the nuclear and granular architecture, leading to extracellular release of NETs. NETs are composed of web-like structures of DNA and antimicrobial proteins such as histones, neutrophil elastase, and myeloperoxidase, and have the ability to entrap and kill microbes [34,35]. NETs are also able to activate coagulation factor XII [8], inactivate anticoagulant TFPI [20], and provide a scaffold for platelet binding and aggregation [36], all of which promote thrombus formation [37]. Blockade of NET activity by DNase or antibodies against DNA-histone complexes results in decreased thrombus formation [20,38,39] and increased microbial dissemination [20,40-42], indicating that NETs play a critical role in immunothrombosis.

### PAMPs and DAMPs

As mentioned above, activated monocytes and neutrophils are two major inducers of immunothrombosis. Therefore, the next question is what activates monocytes and neutrophils to induce immunothrombosis? Pathogen-associated molecular patterns (PAMPs) and damage-associated molecular patterns (DAMPs) might be responsible.

The adaptive immune system composed of T and B lymphocytes monitors non-self antigens using antigen-specific receptors. Self-reactive lymphocytes are deleted early in life, and residual lymphocytes establish a surveillance system for non-self antigens. Although this system is highly specific and effective for non-self antigen elimination, it is not perfect because immune responses against harmless non-self components, such as fetuses or foodstuffs, can be deleterious and should be avoided [43]. Innate immune cells, including monocytes and neutrophils, employ a different surveillance system. They monitor common molecular patterns of microbes (PAMPs) and/or molecules from damaged cells of host origin (DAMPs) using pattern recognition receptors (PRRs) and only activate the adaptive immune system if they detect PAMPs and/or DAMPs. Thus, the innate and adaptive immune systems develop a mutually complementary relationship, and the overall immune system constructs a surveillance system for infectious non-self and/or damaging non-self antigens [43,44].

PAMPs, comprising molecular structures unique to microbes, are subject to innate immune monitoring by the host. For example, cell wall components, such as LPS and β-glucan, or flagellar components, such as flagellin, are recognized as PAMPs, and PAMP detection by PRRs triggers proinflammatory and antimicrobial responses in innate immune cells [45]. PAMP detection also triggers tissue factor expression on monocytes [30,33] and NET release by neutrophils [38], promoting immunothrombosis.

DAMPs are endogenous molecules that are normally found inside cells, unless released by damage. Under normal conditions, DAMPs are hidden from recognition by innate immune cells. However, under conditions of cellular stress or injury, DAMPs can be released into the extracellular space from damaged cells, activating innate immune cells [46]. Prototypical DAMPs include nuclear proteins such as high mobility group box 1 (HMGB1) [47,48] and histones [49], purine metabolites such as ATP [50,51] and uric acid [52,53], and mitochondrial components such as formyl peptides and mitochondrial DNA [54]. Detection of these DAMPs by PRRs, such as Toll-like receptors and inflammasomes, triggers inflammation, which is important for eradication of invading pathogens, clearance of dead cells, and regeneration of damaged tissue [55]. DAMPs also trigger intravascular thrombus formation [50], possibly by inducing tissue factor expression on monocytes [56], elevating tissue factor procoagulant activity [57,58], and promoting platelet aggregation [59].

### Immunothrombosis beyond control

Although immunothrombosis might be important in early host defense against bacterial dissemination, uncontrolled immunothrombosis might be detrimental to the host. Disseminated intravascular coagulation (DIC) occurs in 25%–50% of patients with sepsis and is associated with poor outcomes [12,60]. DIC is characterized by widespread microvascular thrombosis with exhaustion of coagulation factors and platelets [61]. Monocyte-associated tissue factor and neutrophil-derived NETs are predisposing factors for DIC [20,32,38,40,62], indicating that DIC might be an advanced stage of immunothrombosis wherein the immune system is no longer able to restrict PAMP/DAMP spreading and immunothrombosis becomes overwhelmed [13].

As mentioned above, tissue factor-induced coagulation is important for preventing bacterial dissemination [18]. However, excessive coagulopathy can be detrimental [63], and pharmacological inhibition of tissue factor or genetic reduction of tissue factor expression often rescues animals from sepsis-associated lethal coagulopathy [32,33,62]. Similarly, elimination of NETs can decrease organ damage [38,40], although NETs are important for preventing bacterial dissemination [40-42]. These findings support the concept that immunothrombosis can be detrimental if it becomes overwhelmed.

The same is true for DAMPs. Although DAMPs have beneficial roles in immunity and tissue repair [44,64], excessive DAMPs can be detrimental. Serum and plasma HMGB1 levels are elevated in patients with sepsis and/or DIC [65,66] and are correlated with DIC scores. Extracellular HMGB1 stimulates tissue factor expression on monocytes, inhibits protein C activation, and promotes microvascular thrombosis development [56]. Antibodies or antagonists capable of neutralizing HMGB1 reduce organ damage and improve survival of septic mice [65,67,68], indicating that excessive HMGB1 circulating in the blood is detrimental. Plasma histone levels are also elevated in patients with sepsis and DIC [69,70]. Extracellular histones trigger platelet aggregation, fibrin deposition, thrombotic occlusion of microvessels, and exhaustion of coagulation factors and platelets [70]. Extracellular cell-free DNA (cfDNA) also acts as a DAMP [71]. Plasma cfDNA levels are elevated in patients with severe sepsis, especially in non-survivors and have better prognostic utility than Acute Physiology and Chronic Health Evaluation (APACHE) II scores, Multiple Organ Failure Assessment (SOFA) scores, and other biomarkers [72]. The majority of plasma cfDNA is derived from the host [72,73], although some originates from bacteria, fungi, and viruses. cfDNA is the major structural component of NETs, and cfDNA/NETs can promote thrombin generation, in part, through activation of coagulation factor XII [39,74]. Depletion of cfDNA/NETs by DNase treatment impedes early immune responses [75], suggesting that cfDNA-mediated immunothrombosis might be important in early host defense against bacterial dissemination.

In septic conditions, the procoagulant-anticoagulant balance becomes disturbed. While tissue factor- and NET-associated procoagulant activity is increased during sepsis, anticoagulant proteins, such as TM, protein C, and AT, can be severely compromised [60,76-78]. Furthermore, fibrinolysis is attenuated in septic conditions, in part through increased plasminogen activator inhibitor type-1 (PAI-1) release from endothelial cells [60]. Thus, disturbance of the procoagulant-anticoagulant balance, with increases in procoagulant tissue factor and NETs and decreases in anticoagulants and fibrinolytic capacity, is the key feature of sepsis-associated DIC.

### Therapeutic options for DIC

The cornerstone for managing DIC remains the management of the underlying causes, such as sepsis, in most Western countries [61]. Accordingly, there is no mention of DIC in the Surviving Sepsis Campaign guidelines, comprising international guidelines for management of severe sepsis and septic shock [79]. Consequently, anticoagulant drugs might be used for the treatment of sepsis, but not for DIC itself in those countries.

Activated protein C (APC) is a natural anticoagulant that can dampen thrombin generation by inactivating coagulation factors Va and VIIIa (Figure 2). APC also exerts cytoprotective effects, in part through activation of endothelial cell protease-activated receptor 1 [80]. Drotrecogin alfa (activated), a recombinant human APC (rhAPC), used to be the only approved drug associated with significantly improved survival of patients with severe sepsis, based on a large-scale, randomized, double-blind, placebo-controlled, multicenter trial (PROWESS study) [81]. However, the initial success was not replicated in subsequent trials of drotrecogin alfa (activated) in patients with severe sepsis and low risk of death [82], children with severe sepsis [83], and patients with septic shock [84], and this drug has now been withdrawn from the market [85]. Possible reasons for this failure include the increased risk of serious bleeding in the rhAPC group and lower placebo mortality rates compared with the original PROWESS study, making it difficult to demonstrate beneficial effects of rhAPC.

TM is an anticoagulant cofactor that converts thrombin into an APC generator (Figure 2). Because TM is essential for preventing intravascular coagulation [86] and its expression is compromised during sepsis [76], substitution with recombinant human soluble TM (rhsTM) is a promising treatment for patients with sepsis and DIC. Although the anticoagulant action of TM is mainly mediated by APC, rhsTM treatment may have some advantages over rhAPC. First, rhsTM may have less risk of bleeding complications than rhAPC because it is a cofactor and does not act as an anticoagulant when no thrombin exists [87]. Second, the APC-independent actions of rhsTM might confer a benefit. These actions include sequestration of PAMPs [88], DAMPs [68,70,89], and complements [90] through the lectin-like domain of rhsTM [91]. In a randomized, double-blind, parallel-group trial to evaluate DIC resolution rates, rhsTM was significantly superior to heparin for DIC improvement [92]. The 28-day mortality rates were assessed as a secondary endpoint in the study and were 28.0% for the rhsTM group and 34.6% for the heparin group (difference: −6.6%; 95% CI: −24.6 to 11.3) in patients with DIC and infection. Thus, rhsTM has been approved in Japan for treatment of DIC, although further studies are needed to confirm that rhsTM improves clinical outcomes in patients with sepsis-associated DIC. Post-marketing retrospective observational studies suggested that rhsTM therapy might be associated with better outcomes [93-95], and an international, multicenter, randomized, double-blind, placebo-controlled, phase 3 clinical trial for rhsTM is now in progress. Severe sepsis patients with coagulopathy are scheduled to be evaluated in this trial, on the grounds that mortality rates of sepsis patients without organ dysfunction are relatively low and it is thus difficult to evaluate treatment benefits on mortality in these patients, and that patients with coagulopathy might gain greater benefits from anticoagulant therapy [96].

AT is the most abundant anticoagulant protein circulating in the blood. AT is rapidly depleted in the early phases of sepsis through decreased synthesis, increased destruction, and enhanced clearance by thrombin-AT complex (TAT) formation [77,78]. AT has anti-inflammatory and anticoagulant properties. Heparin enhances the anticoagulant activity of AT but may diminish anti-inflammatory effects of AT [97]. The effects of high-dose AT treatment in patients with severe sepsis were investigated in the KyberSept trial, a large-scale, randomized, double-blind, placebo-controlled, phase 3 clinical trial [98]. However, it showed that high-dose AT had no effect on 28-day all-cause mortality and was associated with increased risk of hemorrhage when administered with heparin. There is some evidence to suggest treatment benefits of AT in subgroups of patients not receiving concomitant heparin and complicated with DIC [98-100]. The efficacy and safety of AT need to be confirmed in further studies.

## Conclusions

Immunothrombosis plays an important role in early immune defense against invading pathogens. DIC is considered to be an advanced stage of immunothrombosis, where the immune system is no longer able to restrict PAMP/DAMP spreading and immunothrombosis becomes overwhelmed. In this stage, thrombosis is detrimental because it causes multiple organ failure. Although anticoagulant drugs, such as APC, TM, and AT, are promising options for treatment of sepsis-associated DIC, none of them have been shown to improve the outcomes of patients with sepsis. The key to success may be the selection of proper patients, proper timing, and proper dosages.

## Abbreviations

PAMPs: pathogen-associated molecular patterns
DAMPs: damage-associated molecular patterns
NETs: neutrophil extracellular traps
DIC: disseminated intravascular coagulation
TM: thrombomodulin
TFPI: tissue factor pathway inhibitor
AT: antithrombin
LPS: lipopolysaccharide
PRRs: pattern recognition receptors
HMGB1: high mobility group box 1
cfDNA: cell-free DNA
APACHE II: Acute Physiology and Chronic Health Evaluation II
SOFA: Sequential Organ Failure Assessment
PAI-1: plasminogen activator inhibitor type-1
APC: activated protein C
rhAPC: recombinant human activated protein C
rhsTM: recombinant human soluble thrombomodulin
TAT: thrombin-antithrombin complex

## References

[1] Borissoff JI, Spronk HM, ten Cate H (2011). The hemostatic system as a modulator of atherosclerosis. N Engl J Med.

[2] Jackson SP, Nesbitt WS, Kulkarni S (2003). Signaling events underlying thrombus formation. J Thromb Haemost.

[3] Furie B, Furie BC (2008). Mechanisms of thrombus formation. N Engl J Med.

[4] Ratnoff OD, Colopy JE (1955). A familial hemorrhagic trait associated with a deficiency of a clot-promoting fraction of plasma. J Clin Invest.

[5] Colman RW (2006). Are hemostasis and thrombosis two sides of the same coin?. J Exp Med.

[6] Renne T, Pozgajova M, Gruner S, Schuh K, Pauer HU, Burfeind P, Gailani D, Nieswandt B (2005). Defective thrombus formation in mice lacking coagulation factor XII. J Exp Med.

[7] Kleinschnitz C, Stoll G, Bendszus M, Schuh K, Pauer HU, Burfeind P, Renne C, Gailani D, Nieswandt B, Renne T (2006). Targeting coagulation factor XII provides protection from pathological thrombosis in cerebral ischemia without interfering with hemostasis. J Exp Med.

[8] von Bruhl ML, Stark K, Steinhart A, Chandraratne S, Konrad I, Lorenz M, Khandoga A, Tirniceriu A, Coletti R, Kollnberger M, Byrne RA, Laitinen I, Walch A, Brill A, Pfeiler S, Manukyan D, Braun S, Lange P, Riegger J, Ware J, Eckart A, Haidari S, Rudelius M, Schulz C, Echtler K, Brinkmann V, Schwaiger M, Preissner KT, Wagner DD, Mackman N (2012). Monocytes, neutrophils, and platelets cooperate to initiate and propagate venous thrombosis in mice in vivo. J Exp Med.

[9] Aird WC (2005). Spatial and temporal dynamics of the endothelium. J Thromb Haemost.

[10] Pober JS, Sessa WC (2007). Evolving functions of endothelial cells in inflammation. Nat Rev Immunol.

[11] Esmon CT (1987). The regulation of natural anticoagulant pathways. Science.

[12] Levi M, Ten Cate H (1999). Disseminated intravascular coagulation. N Engl J Med.

[13] Engelmann B, Massberg S (2013). Thrombosis as an intravascular effector of innate immunity. Nat Rev Immunol.

[14] Pfeiler S, Massberg S, Engelmann B (2014). Biological basis and pathological relevance of microvascular thrombosis. Thromb Res.

[15] Delvaeye M, Conway EM (2009). Coagulation and innate immune responses: can we view them separately?. Blood.

[16] Muta T, Iwanaga S (1996). The role of hemolymph coagulation in innate immunity. Curr Opin Immunol.

[17] Cerenius L, Soderhall K (2011). Coagulation in invertebrates. J Innate Immun.

[18] Luo D, Szaba FM, Kummer LW, Plow EF, Mackman N, Gailani D, Smiley ST (2011). Protective roles for fibrin, tissue factor, plasminogen activator inhibitor-1, and thrombin activatable fibrinolysis inhibitor, but not factor XI, during defense against the gram-negative bacterium Yersinia enterocolitica. J Immunol.

[19] Sun H, Wang X, Degen JL, Ginsburg D (2009). Reduced thrombin generation increases host susceptibility to group A streptococcal infection. Blood.

[20] Massberg S, Grahl L, von Bruehl ML, Manukyan D, Pfeiler S, Goosmann C, Brinkmann V, Lorenz M, Bidzhekov K, Khandagale AB, Konrad I, Kennerknecht E, Reges K, Holdenrieder S, Braun S, Reinhardt C, Spannagl M, Preissner KT, Engelmann B (2010). Reciprocal coupling of coagulation and innate immunity via neutrophil serine proteases. Nat Med.

[21] Günther A, Mosavi P, Heinemann S, Ruppert C, Muth H, Markart P, Grimminger F, Walmrath D, Temmesfeld-WollbrÜCk B, Seeger W (2000). Alveolar fibrin formation caused by enhanced procoagulant and depressed fibrinolytic capacities in severe pneumonia. Am J Resp Crit Care Med.

[22] Wong CH, Jenne CN, Petri B, Chrobok NL, Kubes P (2013). Nucleation of platelets with blood-borne pathogens on Kupffer cells precedes other innate immunity and contributes to bacterial clearance. Nat Immunol.

[23] Vieira-de-Abreu A, Campbell RA, Weyrich AS, Zimmerman GA (2012). Platelets: versatile effector cells in hemostasis, inflammation, and the immune continuum. Semin Immunopathol.

[24] Gross AK, Dunn SP, Feola DJ, Martin CA, Charnigo R, Li Z, Abdel-Latif A, Smyth SS (2013). Clopidogrel treatment and the incidence and severity of community acquired pneumonia in a cohort study and meta-analysis of antiplatelet therapy in pneumonia and critical illness. J Thromb Thrombolysis.

[25] Winning J, Reichel J, Eisenhut Y, Hamacher J, Kohl M, Deigner HP, Claus RA, Bauer M, Lösche W (2009). Anti-platelet drugs and outcome in severe infection: clinical impact and underlying mechanisms. Platelets.

[26] Winning J, Neumann J, Kohl M, Claus RA, Reinhart K, Bauer M, Losche W (2010). Antiplatelet drugs and outcome in mixed admissions to an intensive care unit. Crit Care Med.

[27] Loof TG, Morgelin M, Johansson L, Oehmcke S, Olin AI, Dickneite G, Norrby-Teglund A, Theopold U, Herwald H (2011). Coagulation, an ancestral serine protease cascade, exerts a novel function in early immune defense. Blood.

[28] Flick MJ, Du X, Witte DP, Jirouskova M, Soloviev DA, Busuttil SJ, Plow EF, Degen JL (2004). Leukocyte engagement of fibrin(ogen) via the integrin receptor alphaMbeta2/Mac-1 is critical for host inflammatory response in vivo. J Clin Invest.

[29] Frick IM, Akesson P, Herwald H, Morgelin M, Malmsten M, Nagler DK, Bjorck L (2006). The contact system–a novel branch of innate immunity generating antibacterial peptides. EMBO J.

[30] Rivers RP, Hathaway WE, Weston WL (1975). The endotoxin-induced coagulant activity of human monocytes. Br J Haematol.

[31] Egorina EM, Sovershaev MA, Bjørkøy G, Gruber FXE, Olsen JO, Parhami-Seren B, Mann KG, Østerud B (2005). Intracellular and surface distribution of monocyte tissue factor: application to intersubject variability. Arterioscler Thromb Vasc Biol.

[32] Pawlinski R, Pedersen B, Schabbauer G, Tencati M, Holscher T, Boisvert W, Andrade-Gordon P, Frank RD, Mackman N (2004). Role of tissue factor and protease-activated receptors in a mouse model of endotoxemia. Blood.

[33] Pawlinski R, Wang JG, Owens AP, Williams J, Antoniak S, Tencati M, Luther T, Rowley JW, Low EN, Weyrich AS, Mackman N (2010). Hematopoietic and nonhematopoietic cell tissue factor activates the coagulation cascade in endotoxemic mice. Blood.

[34] Brinkmann V, Reichard U, Goosmann C, Fauler B, Uhlemann Y, Weiss DS, Weinrauch Y, Zychlinsky A (2004). Neutrophil extracellular traps kill bacteria. Science.

[35] Brinkmann V, Zychlinsky A (2007). Beneficial suicide: why neutrophils die to make NETs. Nat Rev Microbiol.

[36] Fuchs TA, Brill A, Duerschmied D, Schatzberg D, Monestier M, Myers DD, Wrobleski SK, Wakefield TW, Hartwig JH, Wagner DD (2010). Extracellular DNA traps promote thrombosis. Proc Natl Acad Sci U S A.

[37] Martinod K, Wagner DD (2014). Thrombosis: tangled up in NETs. Blood.

[38] Clark SR, Ma AC, Tavener SA, McDonald B, Goodarzi Z, Kelly MM, Patel KD, Chakrabarti S, McAvoy E, Sinclair GD, Keys EM, Allen-Vercoe E, Devinney R, Doig CJ, Green FH, Kubes P (2007). Platelet TLR4 activates neutrophil extracellular traps to ensnare bacteria in septic blood. Nat Med.

[39] Gould TJ, Vu T, Swystun LL, Dwivedi D, Mai S, Weitz JI, Liaw PC (2014). Neutrophil extracellular traps promote thrombin generation through platelet-dependent and platelet-independent mechanisms. Arterioscler Thromb Vasc Biol.

[40] McDonald B, Urrutia R, Yipp BG, Jenne CN, Kubes P (2012). Intravascular neutrophil extracellular traps capture bacteria from the bloodstream during sepsis. Cell Host Microbe.

[41] Beiter K, Wartha F, Albiger B, Normark S, Zychlinsky A, Henriques-Normark B (2006). An endonuclease allows Streptococcus pneumoniae to escape from neutrophil extracellular traps. Curr Biol.

[42] Walker MJ, Hollands A, Sanderson-Smith ML, Cole JN, Kirk JK, Henningham A, McArthur JD, Dinkla K, Aziz RK, Kansal RG, Simpson AJ, Buchanan JT, Chhatwal GS, Kotb M, Nizet V (2007). DNase Sda1 provides selection pressure for a switch to invasive group A streptococcal infection. Nat Med.

[43] Matzinger P (2002). The danger model: a renewed sense of self. Science.

[44] Kono H, Rock KL (2008). How dying cells alert the immune system to danger. Nat Rev Immunol.

[45] Medzhitov R (2007). Recognition of microorganisms and activation of the immune response. Nature.

[46] Chen GY, Nunez G (2010). Sterile inflammation: sensing and reacting to damage. Nat Rev Immunol.

[47] Scaffidi P, Misteli T, Bianchi ME (2002). Release of chromatin protein HMGB1 by necrotic cells triggers inflammation. Nature.

[48] Rovere-Querini P, Capobianco A, Scaffidi P, Valentinis B, Catalanotti F, Giazzon M, Dumitriu IE, Muller S, Iannacone M, Traversari C, Bianchi ME, Manfredi AA (2004). HMGB1 is an endogenous immune adjuvant released by necrotic cells. EMBO Rep.

[49] Huang H, Evankovich J, Yan W, Nace G, Zhang L, Ross M, Liao X, Billiar T, Xu J, Esmon CT, Tsung A (2011). Endogenous histones function as alarmins in sterile inflammatory liver injury through Toll-like receptor 9 in mice. Hepatology.

[50] McDonald B, Pittman K, Menezes GB, Hirota SA, Slaba I, Waterhouse CC, Beck PL, Muruve DA, Kubes P (2010). Intravascular danger signals guide neutrophils to sites of sterile inflammation. Science.

[51] Trautmann A (2009). Extracellular ATP in the immune system: more than just a “danger signal”. Sci Signal.

[52] Shi Y, Evans JE, Rock KL (2003). Molecular identification of a danger signal that alerts the immune system to dying cells. Nature.

[53] Kono H, Chen CJ, Ontiveros F, Rock KL (2010). Uric acid promotes an acute inflammatory response to sterile cell death in mice. J Clin Invest.

[54] Zhang Q, Raoof M, Chen Y, Sumi Y, Sursal T, Junger W, Brohi K, Itagaki K, Hauser CJ (2010). Circulating mitochondrial DAMPs cause inflammatory responses to injury. Nature.

[55] Zitvogel L, Kepp O, Kroemer G (2010). Decoding cell death signals in inflammation and immunity. Cell.

[56] Ito T, Kawahara K, Nakamura T, Yamada S, Nakamura T, Abeyama K, Hashiguchi T, Maruyama I (2007). High-mobility group box 1 protein promotes development of microvascular thrombosis in rats. J Thromb Haemost.

[57] Furlan-Freguia C, Marchese P, Gruber A, Ruggeri ZM, Ruf W (2011). P2X7 receptor signaling contributes to tissue factor-dependent thrombosis in mice. J Clin Invest.

[58] Reinhardt C, von Bruhl ML, Manukyan D, Grahl L, Lorenz M, Altmann B, Dlugai S, Hess S, Konrad I, Orschiedt L, Mackman N, Ruddock L, Massberg S, Engelmann B (2008). Protein disulfide isomerase acts as an injury response signal that enhances fibrin generation via tissue factor activation. J Clin Invest.

[59] Fuchs TA, Bhandari AA, Wagner DD (2011). Histones induce rapid and profound thrombocytopenia in mice. Blood.

[60] Zeerleder S, Hack CE, Wuillemin WA (2005). Disseminated intravascular coagulation in sepsis. Chest.

[61] Hunt BJ (2014). Bleeding and coagulopathies in critical care. New Engl J Med.

[62] Taylor FB, Chang AC, Peer G, Li A, Ezban M, Hedner U (1998). Active site inhibited factor VIIa (DEGR VIIa) attenuates the coagulant and interleukin-6 and -8, but not tumor necrosis factor, responses of the baboon to LD100 Escherichia coli. Blood.

[63] Osterud B, Flaegstad T (1983). Increased tissue thromboplastin activity in monocytes of patients with meningococcal infection: related to an unfavourable prognosis. Thromb Haemost.

[64] Palumbo R, Sampaolesi M, De Marchis F, Tonlorenzi R, Colombetti S, Mondino A, Cossu G, Bianchi ME (2004). Extracellular HMGB1, a signal of tissue damage, induces mesoangioblast migration and proliferation. J Cell Biol.

[65] Wang H, Bloom O, Zhang M, Vishnubhakat JM, Ombrellino M, Che J, Frazier A, Yang H, Ivanova S, Borovikova L, Manogue KR, Faist E, Abraham E, Andersson J, Andersson U, Molina PE, Abumrad NN, Sama A, Tracey KJ (1999). HMG-1 as a late mediator of endotoxin lethality in mice. Science.

[66] Hatada T, Wada H, Nobori T, Okabayashi K, Maruyama K, Abe Y, Uemoto S, Yamada S, Maruyama I (2005). Plasma concentrations and importance of High Mobility Group Box protein in the prognosis of organ failure in patients with disseminated intravascular coagulation. Thromb Haemost.

[67] Yang H, Ochani M, Li J, Qiang X, Tanovic M, Harris HE, Susarla SM, Ulloa L, Wang H, DiRaimo R, Czura CJ, Wang H, Roth J, Warren HS, Fink MP, Fenton MJ, Andersson U, Tracey KJ (2004). Reversing established sepsis with antagonists of endogenous high-mobility group box 1. Proc Natl Acad Sci U S A.

[68] Abeyama K, Stern DM, Ito Y, Kawahara KI, Yoshimoto Y, Tanaka M, Uchimura T, Ida N, Yamazaki Y, Yamada S, Yamamoto Y, Yamamoto H, Iino S, Taniguchi N, Maruyama I (2005). The N-terminal domain of thrombomodulin sequesters high-mobility group-B1 protein, a novel antiinflammatory mechanism. J Clin Invest.

[69] Xu J, Zhang X, Pelayo R, Monestier M, Ammollo CT, Semeraro F, Taylor FB, Esmon NL, Lupu F, Esmon CT (2009). Extracellular histones are major mediators of death in sepsis. Nat Med.

[70] Nakahara M, Ito T, Kawahara KI, Yamamoto M, Nagasato T, Shrestha B, Yamada S, Miyauchi T, Higuchi K, Takenaka T, Yasuda T, Matsunaga A, Kakihana Y, Hashiguchi T, Kanmura Y, Maruyama I (2013). Recombinant thrombomodulin protects mice against histone-induced lethal thromboembolism. PLoS ONE.

[71] Bamboat ZM, Balachandran VP, Ocuin LM, Obaid H, Plitas G, De Matteo RP (2010). Toll-like receptor 9 inhibition confers protection from liver ischemia-reperfusion injury. Hepatology.

[72] Dwivedi DJ, Toltl LJ, Swystun LL, Pogue J, Liaw KL, Weitz JI, Cook DJ, Fox-Robichaud AE, Liaw PC (2012). Prognostic utility and characterization of cell-free DNA in patients with severe sepsis. Crit Care.

[73] De Vlaminck I, Khush KK, Strehl C, Kohli B, Luikart H, Neff NF, Okamoto J, Snyder TM, Cornfield DN, Nicolls MR, Weill D, Bernstein D, Valantine HA, Quake SR (2013). Temporal response of the human virome to immunosuppression and antiviral therapy. Cell.

[74] Swystun LL, Mukherjee S, Liaw PC (2011). Breast cancer chemotherapy induces the release of cell-free DNA, a novel procoagulant stimulus. J Thromb Haemost.

[75] Meng W, Paunel-Gorgulu A, Flohe S, Hoffmann A, Witte I, Mackenzie C, Baldus SE, Windolf J, Logters TT (2012). Depletion of neutrophil extracellular traps in vivo results in hypersusceptibility to polymicrobial sepsis in mice. Crit Care.

[76] Faust SN, Levin M, Harrison OB, Goldin RD, Lockhart MS, Kondaveeti S, Laszik Z, Esmon CT, Heyderman RS (2001). Dysfunction of endothelial protein C activation in severe meningococcal sepsis. N Engl J Med.

[77] Niessen RW, Lamping RJ, Jansen PM, Prins MH, Peters M, Taylor FB, de Vijlder JJ, ten Cate JW, Hack CE, Sturk A (1997). Antithrombin acts as a negative acute phase protein as established with studies on HepG2 cells and in baboons. Thromb Haemost.

[78] Opal SM (2000). Therapeutic rationale for antithrombin III in sepsis. Crit Care Med.

[79] Dellinger RP, Levy MM, Rhodes A, Annane D, Gerlach H, Opal SM, Sevransky JE, Sprung CL, Douglas IS, Jaeschke R, Osborn TM, Nunnally ME, Townsend SR, Reinhart K, Kleinpell RM, Angus DC, Deutschman CS, Machado FR, Rubenfeld GD, Webb SA, Beale RJ, Vincent JL, Moreno R, Surviving Sepsis Campaign Guidelines Committee including the Pediatric S (2013). Surviving sepsis campaign: international guidelines for management of severe sepsis and septic shock: 2012. Crit Care Med.

[80] Riewald M, Petrovan RJ, Donner A, Mueller BM, Ruf W (2002). Activation of endothelial cell protease activated receptor 1 by the protein C pathway. Science.

[81] Bernard GR, Vincent JL, Laterre PF, LaRosa SP, Dhainaut JF, Lopez-Rodriguez A, Steingrub JS, Garber GE, Helterbrand JD, Ely EW, Fisher CJ (2001). Efficacy and safety of recombinant human activated protein C for severe sepsis. New Engl J Med.

[82] Abraham E, Laterre PF, Garg R, Levy H, Talwar D, Trzaskoma BL, François B, Guy JS, Brückmann M, Rea-Neto Á, Rossaint R, Perrotin D, Sablotzki A, Arkins N, Utterback BG, Macias WL (2005). Drotrecogin alfa (activated) for adults with severe sepsis and a low risk of death. New Engl J Med.

[83] Nadel S, Goldstein B, Williams MD, Dalton H, Peters M, Macias WL, Abd-Allah SA, Levy H, Angle R, Wang D, Sundin DP, Giroir B (2007). Drotrecogin alfa (activated) in children with severe sepsis: a multicentre phase III randomised controlled trial. Lancet.

[84] Ranieri VM, Thompson BT, Barie PS, Dhainaut JF, Douglas IS, Finfer S, Gårdlund B, Marshall JC, Rhodes A, Artigas A, Payen D, Tenhunen J, Al-Khalidi HR, Thompson V, Janes J, Macias WL, Vangerow B, Williams MD (2012). Drotrecogin alfa (activated) in adults with septic shock. New Engl J Med.

[85] Mitka M (2011). Drug for severe sepsis is withdrawn from market, fails to reduce mortality. JAMA.

[86] Isermann B, Hendrickson SB, Zogg M, Wing M, Cummiskey M, Kisanuki YY, Yanagisawa M, Weiler H (2001). Endothelium-specific loss of murine thrombomodulin disrupts the protein C anticoagulant pathway and causes juvenile-onset thrombosis. J Clin Invest.

[87] Ito T, Maruyama I (2011). Thrombomodulin: protectorate God of the vasculature in thrombosis and inflammation. J Thromb Haemost.

[88] Shi CS, Shi GY, Hsiao SM, Kao YC, Kuo KL, Ma CY, Kuo CH, Chang BI, Chang CF, Lin CH, Wong CH, Wu HL (2008). Lectin-like domain of thrombomodulin binds to its specific ligand Lewis Y antigen and neutralizes lipopolysaccharide-induced inflammatory response. Blood.

[89] Ito T, Kawahara K, Okamoto K, Yamada S, Yasuda M, Imaizumi H, Nawa Y, Meng X, Shrestha B, Hashiguchi T, Maruyama I (2008). Proteolytic cleavage of high mobility group box 1 protein by thrombin-thrombomodulin complexes. Arterioscler Thromb Vasc Biol.

[90] Delvaeye M, Noris M, De Vriese A, Esmon CT, Esmon NL, Ferrell G, Del-Favero J, Plaisance S, Claes B, Lambrechts D, Zoja C, Remuzzi G, Conway EM (2009). Thrombomodulin mutations in atypical hemolytic-uremic syndrome. N Engl J Med.

[91] Conway EM, Van de Wouwer M, Pollefeyt S, Jurk K, Van Aken H, De Vriese A, Weitz JI, Weiler H, Hellings PW, Schaeffer P, Herbert JM, Collen D, Theilmeier G (2002). The lectin-like domain of thrombomodulin confers protection from neutrophil-mediated tissue damage by suppressing adhesion molecule expression via nuclear factor kappaB and mitogen-activated protein kinase pathways. J Exp Med.

[92] Saito H, Maruyama I, Shimazaki S, Yamamoto Y, Aikawa N, Ohno R, Hirayama A, Matsuda T, Asakura H, Nakashima M, Aoki N (2007). Efficacy and safety of recombinant human soluble thrombomodulin (ART-123) in disseminated intravascular coagulation: results of a phase III, randomized, double-blind clinical trial. J Thromb Haemost.

[93] Ogawa Y, Yamakawa K, Ogura H, Kiguchi T, Mohri T, Nakamori Y, Kuwagata Y, Shimazu T, Hamasaki T, Fujimi S (2012). Recombinant human soluble thrombomodulin improves mortality and respiratory dysfunction in patients with severe sepsis. J Trauma Acute Care Surg.

[94] Yamakawa K, Fujimi S, Mohri T, Matsuda H, Nakamori Y, Hirose T, Tasaki O, Ogura H, Kuwagata Y, Hamasaki T, Shimazu T (2011). Treatment effects of recombinant human soluble thrombomodulin in patients with severe sepsis: a historical control study. Crit Care.

[95] Yamakawa K, Ogura H, Fujimi S, Morikawa M, Ogawa Y, Mohri T, Nakamori Y, Inoue Y, Kuwagata Y, Tanaka H, Hamasaki T, Shimazu T (2013). Recombinant human soluble thrombomodulin in sepsis-induced disseminated intravascular coagulation: a multicenter propensity score analysis. Intensive Care Med.

[96] Vincent JL, Ramesh MK, Ernest D, Larosa SP, Pachl J, Aikawa N, Hoste E, Levy H, Hirman J, Levi M, Daga M, Kutsogiannis DJ, Crowther M, Bernard GR, Devriendt J, Puigserver JV, Blanzaco DU, Esmon CT, Parrillo JE, Guzzi L, Henderson SJ, Pothirat C, Mehta P, Fareed J, Talwar D, Tsuruta K, Gorelick KJ, Osawa Y, Kaul I (2013). A randomized, double-blind, placebo-controlled, phase 2b study to evaluate the safety and efficacy of recombinant human soluble thrombomodulin, ART-123, in patients with sepsis and suspected disseminated intravascular coagulation. Crit Care Med.

[97] Hoffmann JN, Vollmar B, Laschke MW, Inthorn D, Kaneider NC, Dunzendorfer S, Wiedermann CJ, Romisch J, Schildberg FW, Menger MD (2002). Adverse effect of heparin on antithrombin action during endotoxemia: microhemodynamic and cellular mechanisms. Thromb Haemost.

[98] Warren BL, Eid A, Singer P, Pillay SS, Carl P, Novak I, Chalupa P, Atherstone A, Penzes I, Kubler A, Knaub S, Keinecke HO, Heinrichs H, Schindel F, Juers M, Bone RC, Opal SM (2001). Caring for the critically ill patient. High-dose antithrombin III in severe sepsis: a randomized controlled trial. JAMA.

[99] Kienast J, Juers M, Wiedermann CJ, Hoffmann JN, Ostermann H, Strauss R, Keinecke HO, Warren BL, Opal SM (2006). Treatment effects of high-dose antithrombin without concomitant heparin in patients with severe sepsis with or without disseminated intravascular coagulation. J Thromb Haemost.

[100] Wiedermann CJ, Hoffmann JN, Juers M, Ostermann H, Kienast J, Briegel J, Strauss R, Keinecke HO, Warren BL, Opal SM (2006). High-dose antithrombin III in the treatment of severe sepsis in patients with a high risk of death: efficacy and safety. Crit Care Med.

